# Pathogenicity and virulence of *Pseudomonas aeruginosa*: Recent advances and under-investigated topics

**DOI:** 10.1080/21505594.2025.2503430

**Published:** 2025-05-12

**Authors:** Jemima Swain, Isabel Askenasy, Rahan Rudland Nazeer, Pok-Man Ho, Edoardo Labrini, Leonardo Mancini, Qingqing Xu, Franziska Hollendung, Isabella Sheldon, Camilla Dickson, Amelie Welch, Adam Agbamu, Camilla Godlee, Martin Welch

**Affiliations:** Department of Biochemistry, Cambridge University, Cambridge, UK

**Keywords:** *Pseudomonas aeruginosa*, virulence, polymicrobial, host susceptibility, biofilms, quorum sensing

## Abstract

*Pseudomonas aeruginosa* is a model for the study of quorum sensing, protein secretion, and biofilm formation. Consequently, it has become one of the most intensely reviewed pathogens, with many excellent articles in the current literature focusing on these aspects of the organism’s biology. Here, though, we aim to take a slightly different approach and consider some less well appreciated (but nonetheless important) factors that affect *P. aeruginosa* virulence. We start by reminding the reader of the global importance of *P. aeruginosa* infection and that the “virulome” is very niche–specific. Overlooked but obvious questions such as “what prevents secreted protein products from being digested by co-secreted proteases?” are discussed, and we suggest how the nutritional preference(s) of the organism might dictate its environmental reservoirs. Recent studies identifying host genes associated with genetic predisposition towards *P. aeruginosa* infection (and even infection by specific *P. aeruginosa* strains) and the role(s) of intracellular *P. aeruginosa* are introduced. We also discuss the fact that virulence is a high-risk strategy and touch on how expression of the two main classes of virulence factors is regulated. A particular focus is on recent findings highlighting how nutritional status and metabolism are as important as quorum sensing in terms of their impact on virulence, and how co-habiting microbial species at the infection site impact on *P. aeruginosa* virulence (and *vice versa*). It is our view that investigation of these issues is likely to dominate many aspects of research into this WHO-designated priority pathogen over the next decade.

## The pathogen in context

In 2019, the GRAM study [1] reported that of the 7.7 million deaths caused by bacterial infection worldwide, over half (54%) were attributable to just five species; *Staphylococcus aureus* (1.1 million deaths), *Escherichia coli* (950,000 deaths), *Streptococcus pneumoniae* (829,000 deaths), *Klebsiella pneumoniae* (790,000 deaths), and *Pseudomonas aeruginosa* (559,000 deaths). Of these, and because of its notorious (and ever-growing) recalcitrance to therapeutic intervention, *P. aeruginosa* is particularly dreaded among the clinical community. Indeed, around 7% of all healthcare-associated infections are linked to the organism, with incidence particularly high in intensive care units (up to 23%).

**Figure 1.**
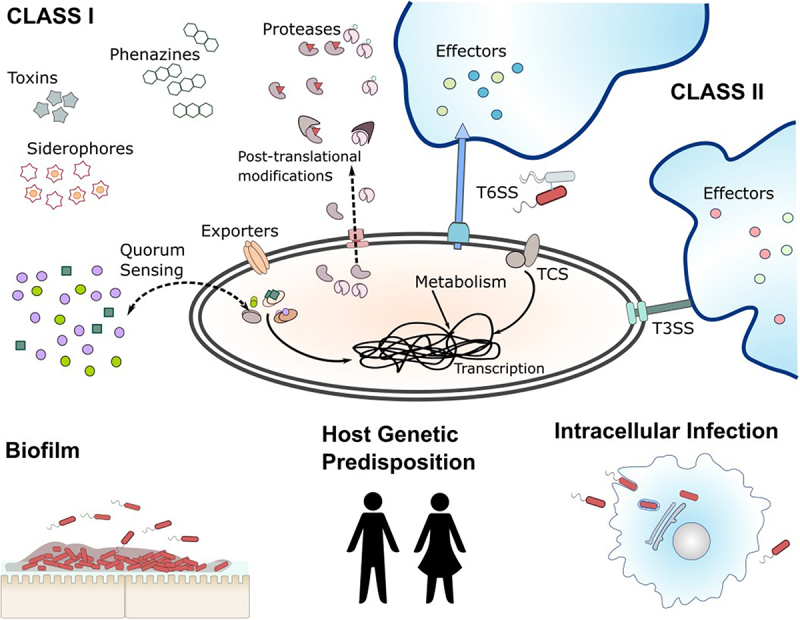
Summary of the key topic areas discussed in this review. The figure contrasts the activities of Class I secreted virulence factors such as proteases, phospholipases, toxins, and secondary metabolites (e.g. phenazines and siderophores) with Class II factors delivered through the Type III and Type VI injectisomes. The distinct pathways regulating expression of Class I and Class II virulence factors through quorum sensing and two-component signalling systems, respectively, is indicated. The figure also highlights the importance of post-translational modification of secreted Class I factors (to prevent autodigestion by co-secreted proteases) as well as the intracellular and biofilm-associated lifestyles of *P. aeruginosa*. Although biofilm-associated cells are traditionally considered to be less virulent that their planktonic counterparts, they are not avirulent, and are now known to secrete a distinct spectrum of virulence factors. The integral, but under-explored, link between virulence and metabolism is indicated, as is emerging evidence suggesting that genetic factors predispose the host towards *P. aeruginosa* infection.

A Gram-negative gammaproteobacterium, in 2024, *P. aeruginosa* was designated by the World Health Organization (WHO) as a priority pathogen (“downgraded” slightly from its designation as a *critical* priority pathogen in the 2017 assessment) [2,3]. The reasons for this somewhat dubious accolade are its ubiquity in the built environment, its ability to infect most tissue types, its extensive repertoire of intrinsic and acquired antibiotic resistance mechanisms, and its remarkable arsenal of virulence factors. The current commentary focuses on the latter, with a particular emphasis on the flexibility of its pathogenic toolkit and the different strategies that the organism uses to deploy this. We also want to touch upon certain aspects of *P. aeruginosa* pathogenicity that are not normally covered in most reviews on the topic, but which are increasingly being recognized as critical in many infections (summarized in Figure 1).

## *P. aeruginosa* and the built environment

While often thought of as being an archetypal pathogen, it is not; *P. aeruginosa* is very much an opportunist rather than a “professional killer” [4,5]. Indeed, its reputation as a pathogen probably has as much to do with its fondness for colonizing anthropic niches as it does with its innate pathogenicity. *P. aeruginosa* is significantly more common in human-associated environments than in environments that experience little or no human contact. For example, it is more abundant in compost than in uncontaminated soil, and in drains and sewers than in pristine rivers [6]. An alarming consequence of this affinity for the built environment is the prevalence of *P. aeruginosa* in healthcare settings (notably, in hospital drains [7]). Whether these drains serve as reservoirs for nosocomial infection is not yet known [8].

It is not yet known why *P. aeruginosa* has such a proclivity for the urban environment, although reservoirs of the organism frequently have a common unifying feature: they involve stagnant warm water that has been in contact with the human body (e.g. shower drains, washroom U-tubes, hot tubs, etc.). One explanation for this may lie in the organism’s strong dietary preference for fatty acids [9–13]. Given that humans are covered in skin-moisturizing fatty acid derivatives (collectively known as sebum), and given that most of us helpfully solubilize these oils on a daily basis (ironically, with the help of a bar of solidified sodium stearate), it should come as no surprise that *P. aeruginosa* feels very much at home in the bathroom plumbing.

## Infection scenarios

It used to be thought that *P. aeruginosa*-associated infections are rare in non-human mammals, although reports of otitis media in dogs, wound infections in cattle, and mastitis in sheep, etc. are widespread [14–17], so this may simply be a reporting/testing artefact or a reflection of its relative rarity outside of the urban environment. In humans, *P. aeruginosa* is generally not much of a threat to healthy, immune-competent subjects. However, given the opportunity, e.g. a breach in the skin barrier, *P. aeruginosa* will invade and colonize almost any tissue. Lower respiratory tract infections are common, especially in people with genetic or lifestyle-associated predispositions. These include people with mutations in the *CFTR* leading to cystic fibrosis (CF), or pollution/smoke-induced lung damage leading to COPD. *P. aeruginosa* is also strongly associated with infections in the eye, diabetic ulcers, and post-operative wounds. Although these infection sites/types have been known about for decades, depressingly, trends have barely changed since the early 1980s [18].

In the UK, *P. aeruginosa* causes around 10% of all catheter-associated UTIs, 10% of all ventilator-associated pneumonias, and 5% of all surgical site infections [8]. The immune dysfunction that often accompanies diabetic hyperglycaemia also pre-disposes this cadre of patients to ulcerous infections, many of which involve *P. aeruginosa* [19–21]. The numbers for all of these infection types have risen considerably in lower-middle income (LMIC) nations. The reasons for this are not yet clear; inadequate hygiene, poor antimicrobial stewardship, and inadequate appreciation of infection control outside of the clinic are possible drivers [22–24].

Over past decades, due to better implementation of infection control measures, *P. aeruginosa* was becoming less common in burn units. However, this welcome trend has now been reversed and between 2008 and 2018, *P. aeruginosa* was responsible for 57% of all wound infections in burn units [8,25]. Interestingly, the apparent predilection of *P. aeruginosa* for burn wounds is probably due to two factors. First, the chemical composition of burn wound exudates actively disfavours the growth of other common opportunists, so *P. aeruginosa* “wins out” by default [26]. Second, burn wound exudates (and infected human plasma [27]) are known to stimulate quorum sensing, and consequently, also the production of certain virulence factors such as proteases, pyocyanin, and siderophores [26].

## Genetic predispositions towards *P. aeruginosa* infection

These need not be limited to the most obvious defects such as loss-of-function mutations in *CFTR*, although *CFTR* mutations remain the most significant genetic drivers of airway susceptibility to *P. aeruginosa* infection that we know about. Studies in mice [28], as well as genome-wide association studies (GWAS) in humans have revealed that a welter of additional genes with diverse functions (including an amino acid transporter (*SLC6A14*), a Na^+^/H^+^ antiporter (*SLC9A3*), and a mannose-binding lectin (*MBL2*), among others) can modify CFTR-dependent susceptibility to *P. aeruginosa* [29,30].

Interestingly, these host-associated factors influence not only susceptibility to the species *per se*; they also influence susceptibility to specific genetic variants (such as mucoid strains) of the species, the time to first airway infection by *P. aeruginosa*, and the age of onset of chronic infection [31]. Presumably, the same or similar genetic modifiers may also affect susceptibility in the non-CF population too, although we are not aware of any comparable GWAS studies carried out on, e.g. the COPD population. Given the prevalence of COPD (projected to be 600 million cases worldwide by 2025 [32]), this would be worthwhile, especially if prophylaxis can be put in place to minimize the considerable on-costs to already stretched healthcare systems of treating full-blown *P. aeruginosa* airway infections.

## One size does not fit all: secreted *P. aeruginosa* virulence factors display substantial strain-to-strain variation

Although we are currently going through a re-appraisal of what defines a pathogen [4,33,34], virulence is a somewhat easier term to define since it describes the relative capacity of the microbe to colonize the host, cause damage to the host, or evade the host immune response. Between-strain differences in virulence are common. This is nicely illustrated by PAO1 and UCBPP-PA14 (more commonly referred to as PA14); the two most widely used laboratory strains of *P. aeruginosa*, which are, respectively, moderately and hyper-virulent [35]. The greater virulence of PA14 is due, in part, to a mutation in *ladS,* which results in increased Type III Secretion System activity and consequently, increased cytotoxicity towards the host [36]. Inter-strain variation is also widely observed between clinical *P. aeruginosa* isolates, often a result of differences in accessory genome content [37,38]. Most virulence factors are secreted products, and it turns out that whereas the intracellular proteome of *P. aeruginosa* isolates is highly similar across different clades (isolates obtained from different sources), the secretome is often strain-specific [39]. This variation in virulence factor secretion may reflect niche specialization on the part of the organism, and raises the question of whether any such strains should be considered “wild type” [40]. Similar niche-specific adaptations were observed when carbon fluxes (especially in the glyoxylate shunt and TCA cycle) were analysed across different strains [41], and the most recent evidence suggests that some *P. aeruginosa* clades become so specialized that transmission between different patients with CF becomes constrained [42]. It is also worth noting at this juncture that a common feature among many late stage CF-adapted isolates is that they become attenuated in the production of virulence factors [43].

## Two main types of virulence factor

*P. aeruginosa* is not an inherently “malevolent” organism; it secretes virulence factors for just three main reasons, (i) to generate nutrients for growth through digestion of host tissue, (ii) to protect this nutritional windfall from opportunistic freeloaders in the neighbourhood, and (iii) to protect itself from immune cell clearance or predation. The virulence factors themselves fall into two broad categories;

- Class I. Tissue-degrading enzymes and toxins that are secreted directly into the surrounding milieu, such as the proteases (e.g. LasA, LasB, AprA, PrpL), phospholipases (PlcB), toxins (ToxA), and small molecules (phenazines, cyanide, rhamnolipids etc.) [44]. The expression of most Class I virulence factors is under the control of the quorum sensing system(s) of *P. aeruginosa*.
- Class II. Tissue-degrading factors that are directly delivered into target cells through specialized injectisomes (the Type III Secretion (T3S) and Type VI Secretion (T6S) systems) [45]. Current evidence suggests that the expression of Class II factors is primarily controlled by inputs from two-component signalling systems that converge at GacS.

Although collectively Class I virulence factors can wreak immense damage on the host, their impact pales compared with the Class II virulence effectors. The latter are the “nuclear weapons” in the *P. aeruginosa* arsenal and, molecule-for-molecule, are far more destructive than any of the Class I factors. In this regard, most “professional” pathogens (if such entities exist – for convenience here, we retain the term) such as *Yersinia pestis* invariably deploy injectisomes as their primary weapon of choice [46,47].

The synthesis and secretion of Class I factors are primarily (but not entirely) regulated by a cell density sensing mechanism known as quorum sensing (QS, reviewed extensively elsewhere [48]), whereas synthesis of the Class II determinants is mostly – but not entirely – QS-independent. Instead, the synthesis of Class II factors is primarily regulated by two-component signalling systems, such as the RetS/LadS/GacS/PA1611 pathway [49–51]. Note the caveats here though; most regulatory systems are inter-linked, and while these statements are broadly correct, it is also true to say that there are circumstances when (for example) the T3S system displays a degree of regulation by QS [52].

## Virulence as a high-risk strategy

It is worth recalling at this point that the production and secretion of Class I virulence factors are resource intensive for the organism. Moreover, it is no coincidence that these factors are secreted just when the organism is running out of nutrients, upon entry into the stationary phase. [It has been known for many years now that adding QS signals early in the growth curve does not advance virulence factor production.] This is because the production of secreted virulence factors is simply a means-to-an-end for securing additional nutrients; it is *a nutrient limitation response strategy*. This notion is further reinforced by the recent finding that virulence factor elaboration is strongly influenced by the stress alarmone, (p)ppGpp, which accumulates when cells run short of nutrient [53]. However, the strategy is not risk-free. Once Class I virulence factors have been secreted, the organism has very little control over their destiny; if they hit a target tissue and degrade this to yield nutrients, then bingo, the producing cells see a payoff. Equally, the virulence factors may diffuse harmlessly away and not benefit the producing cells at all. In this respect, the elaboration of Class I factors represents a distinct metabolic gamble – but one that clearly works, since it has obviously been selected during the course of evolution.

## Cheats exploit Class I factors

As with all “public goods,” secreted Class I virulence factors can be exploited by co-habiting organisms in the infection niche, creating fertile ground for the appearance of non-producing “cheats” [54]. This is all the more of a problem in *P. aeruginosa*, since mutants defective in QS lose the ability to produce most Class I factors. Such mutants can sup off the resources generated by the investments of their QS^+^ counterpartners while making no such investments themselves. This gives QS^−^ mutants in such mixed populations a distinct growth advantage. Not surprisingly, QS^−^ mutants (e.g., defective in the *lasR* “master regulator” of QS) are common among CF isolates and have been postulated to arise as a consequence of the relative growth advantage associated with cheating. Mitigating, common sense dictates that it would be detrimental for such cheats to sweep through the population since, as they do so, this impacts both the QS^+^ and QS^−^ variants, leading to the so-called “tragedy of the commons” [55].

It seems that the theoretical growth advantage of cheats may be held in check by “policing” mechanisms [56–58]. Here, and through the simple expedient of ensuring that QS also regulates critical intracellular resources (so-called “private goods” such as nucleoside hydrolase, Nuh) or toxins that differentially “punish” cheats (such as cyanide), the fitness advantage of cheats is constrained. We also note that environmental conditions also impact on the stability of cheating populations themselves [59]. Interestingly, and somewhat counter-intuitively, when cheats with different traits co-habit, the overall population can become stabilized. For example, populations containing mixtures of Δ*pvdS* mutants (defective in siderophore production) and Δ*lasR* mutants are more stable than mixtures containing the wild type and either mutant alone [60]. Given that *P. aeruginosa* populations at many chronic infection sites are clonally derived but heterogenous, this raises the question of whether such intra-species diversity is selected precisely because it serves to maintain wider population stability.

## Class II factor-dependent immunosuppression is a cheatable trait

The outcome of Class II virulence factor activity (lysis of host cells and nutrient release) is not the same as the outcome of Class I activity; the cell lysis elicited by Class II factors sets the stage for subsequent activity of Class I factors (which digest the released macromolecules, thereby generating the necessary biosynthetic building blocks that are actually utilized by the pathogen). Nevertheless, and although their fundamental mode of action is entirely different, Class II virulence factors (such as the T3S system) are also a target for cheating. On the surface, this might be rationalized by the argument that nearby T3S^−^ cells can utilize the remains of host cells lysed by T3S^+^ cells, thereby benefitting from the inadvertent largesse of the latter. However, it turns out that although “cheats” defective in the T3S system are common in healthy hosts, they fail to thrive in immune-deficient hosts, suggesting that T3S system-mediated immunosuppression is the most likely “public good” exploited by such cheats [61,62].

## The challenges of life in a self-produced “proteolytic soup”

*P. aeruginosa* faces a major self-inflicted chemical challenge; many Class I factors are potent proteases, such as elastase (LasB), staphylolytic protease (LasA), alkaline protease (AprA), protease IV (PIV), small protease (PASP), and aminopeptidase (PaAP) (recently reviewed by [63]). The accumulation of these proteases in the cultural milieu presents the organism with a problem; how to avoid autodigestion.

One likely solution to this problem is that secreted Class I factors (and, probably, also surface-exposed outer membrane proteins too) are post-translationally modified en-route out of the cell, such that they become resistant to autodigestion (reviewed in [64,65]). Evidence for such modifications used to be clear when 2D gel-based proteomic analyses were in vogue; most Class I enzymes on these gels manifest as “charge trains” (i.e. as spots with different PI values but similar molecular masses) indicative of post-translational modification [66–68]. The intracellular *P. aeruginosa* proteome shows very little evidence of such charge trains [68]. Detailed analyses of key charge trains reveal that the proteins in the spots are modified by methylation and acetylation, and that these modifications map to the surface of the proteins. Furthermore, the degree of modification increases as each charge train is traversed [69]. Presumably, these modifications may act to protect the secreted proteins from digestion by other proteases in the secretome.

## Top-down nutrient sensing controls Class I virulence factor production in *P. aeruginosa*

Although a bewildering array of different inputs have been shown to impinge on the production of Class I virulence factors by *P. aeruginosa* (reviewed in [70]), regulation remains very “top down,” with just two hierarchical pathways – global nutrient sensing and quorum sensing – apparently playing a key role. In *P. aeruginosa*, the expression of the QS “master regulators,” *lasR* and *rhlR*, is subservient to nutrient sensing [53]. This makes good economic sense; after all, why secrete metabolically costly virulence factors if the culture is still awash with nutrients? In the light of these findings, it is not surprising that the accumulation of QS signals in the culture was recently shown to be strongly dependent on both the growth rate and the population cell density [71].

Whereas cell numbers are titrated by the QS system (an *inter*-cellular signalling mechanism), nutritional status is titrated through the *intra*-cellular alarmone, (p)ppGpp. (P)ppGpp is made of two intracellular proteins; ribosome-associated RelA (which senses the presence of uncharged tRNAs entering the A-site of the ribosome), and SpoT (which senses limitations of phosphorus, nitrogen, sulfur, and iron in the cell) [72]. (P)ppGpp is thought to primarily act by binding and reprogramming RNA polymerase directly, although in *E. coli*, the molecule has been recently shown to interface more directly with metabolism [73].

The pathway linking (p)ppGpp to QS and virulence has not yet been elucidated, although mutations in a transcriptional regulator (*mexT*) are known to enable the (p)ppGpp-dependence of virulence factor production to be bypassed and QS to be restored [53]. Mutations in *mexT* also enable virulence to be restored in *lasR* mutants, indicating that it likely sits downstream of both (p)ppGpp and QS [74–78]. Interestingly, in another Gram-negative secretor, the enteric phytopathogen *Pectobacterium atrosepticum*, exoenzyme production is also dependent on both QS and (p)ppGpp. However, here the pathways are not hierarchical, but rather form a *coincidence circuit* which converges at RsmA [79]. It seems that although both of these “QS-regulated secretors” are responsive to the same demands (nutrient limitation and population cell density) they are “wired up” in very different ways.

## Class II virulence factor production is regulated by specific extracellular cues

Whereas Class I virulence factors are controlled by nutrient availability and QS, Class II factors appear to be primarily (but again, not exclusively) regulated by lifestyle choices. These, in turn, are controlled by signalling through the RetS/LadS/GacS/PA1611 cascade [80]. Moreover, Class II injectisomes (the T3S and T6S systems) are normally reciprocally regulated, such that T3S is “off” in biofilms, whereas T6S is “on,” and *vice versa* in planktonic cultures [81,82]. Again, this type of “black-and-white” relationship needs to be considered with caution since there are exceptions to this rule [83] and examples of mutations that lead to dysregulated co-expression of both secretion systems (e.g., [84]). GacS, RetS, LadS, and PA1611 are all membrane-associated “hybrid” sensor histidine kinases. At the heart of the pathway lies GacS. When activated, GacS phosphorylates GacA, which, in turn, stimulates the expression of two small RNAs, rsmZ and rsmY. These RNAs bind and sequester the small protein RsmA (which, in spite of its size, is a global regulator of gene expression), thereby promoting the expression of biofilm-associated genes, including the T6S system [82].

The discussion above begs the question of what activates GacS? It turns out that although GacS *may* sense extracellular ligands directly (though the evidence for this is sparse), its activity appears to be primarily regulated either through transphosphorylation from LadS, or *via* the formation of inactive heterodimers with RetS [80,85]. LadS and RetS both contain periplasmic DISMED-2 domains and are thought to sense Ca^2+^ and airway mucin-derived glycans, respectively [86,87], although quite how specific those cues are is not yet clear. To complicate things further, PA1611 is known to form heterodimers with RetS, potentially antagonizing the activity of the latter (and concomitantly, reactivating signalling through GacS) [88,89]. However, and to emphasize the point made earlier again, there is considerable regulatory crosstalk between the Gac signalling pathways and the pathways regulating Class I virulence factor production. For example, rsmY and rsmZ are known to impinge on the production of *N*-acylhomoserine lactone QS signals (and *vice versa*) [90], and the cAMP-responsive transcription factor Vfr, regulates both T3S (Class II) and QS (Class I) [91–93].

## Production of Class II virulence factors is fine-tuned by cyclic-di-GMP signalling

In addition to the signalling pathways just described, Class II factor production is also regulated by a network of proteins that interact with/synthesize/degrade a second messenger called cyclic di-GMP (c-di-GMP, *bis*-(3′-5′)-cyclic dimeric guanosine monophosphate). As noted by Hall and Lee, who applied a “molecular” version of Koch’s principles to define virulence, the fact that (i) c-di-GMP is made during infection, (ii) that c-di-GMP receptor proteins are required for infection, and (iii) that the processes regulated by c-di-GMP are required for infection, all strongly indicates that this signalling molecule plays a key role in pathogenesis [94].

C-di-GMP is made of diguanylate cyclases, which are characterized by the presence of a GGDEF amino acid motif, and the molecule is broken down by specific phosphodiesterases containing the amino acid motif EAL or HD-GYP [94]. Once synthesized, c-di-GMP binds to a variety of targets in the cell, including primarily transcription factors, such as Alg44, FleQ, PelD, and HapZ, and also RNA riboswitches, thereby altering their activity. The *P. aeruginosa* PAO1 genome encodes no fewer than 17 proteins containing GGDEF domains, 5 proteins containing EAL domains, and 16 proteins containing both GGDEF and EAL domains [94]. Most of these are “output domains” located in signalling complexes that titrate a wide range of environmental inputs – usually *via* membrane-associated sensor histidine kinases. Generally, c-di-GMP is thought to act at a level lower down the regulatory hierarchy than the RetS/LadS/GacS/PA1611 pathway, but is nonetheless absolutely essential for the expression of many biofilm-associated virulence factors (see below).

## Metabolism has its say

So far, we have not discussed whether Class II virulence factor (injectisome) production is affected by nutrient limitation. It is, but *via* a very different mechanism compared with the Class I virulence factors. Whereas (p)ppGpp levels – whose level is determined by the availability of several key nutrients, such as amino acids – modulate the synthesis of Class I virulence factors [53,95,96], it turns out that Class II factors (injectisomes) are regulated by central metabolites or metabolic fluxes. Mechanistically, we do not know why this is, but it is clear that certain “metabolic nodes” impinge strongly on e.g., T3S. For example, when the glyoxylate shunt enzyme, *iso*citrate lyase (ICL, encoded by *aceA*) is inactivated by mutation or inhibition, T3S is severely diminished [83,97].

Interestingly, Nature appears to have recognized the glyoxylate shunt as a metabolic weak point long ago; activated pro-inflammatory macrophages secrete copious quantities of an ICL inhibitor, itaconate, synthesized by the product of the LPS-inducible gene *IRG1* [98,99]. These observations reinforce the notion that the glyoxylate shunt enzymes [100,101] may be a good target for anti-microbial intervention, since their inhibition should simultaneously diminish metabolic fitness at the infection site, and also virulence (T3S) [102,103]. However, *P. aeruginosa* has apparently co-evolved to utilize macrophage-derived itaconate as a carbon source (and concomitantly, promote biofilm formation) [104]. Moreover, itaconate turns out to be a potent immune-modulatory molecule in its own right, and much remains to be understood about its mode-of-action [105]. That metabolism *should* impinge on virulence is hardly surprising, given that it is a direct titrator of nutrient availability in the cell.

## Intracellular *Pseudomonas aeruginosa*

Although traditionally thought of as being an archetypal extracellular pathogen, a less well-appreciated side of *P. aeruginosa* is that it also has the capacity to reside inside host cells, including lung epithelial cells, corneal cells, and urinary tract cells *in vitro*. Indeed, in an airway epithelial cell infection model, *P. aeruginosa* was shown to survive and replicate intracellularly for up to 5 days [106]. Importantly, intracellular *P. aeruginosa* has recently been identified in the ciliated airway epithelial cells of lung explants from CF patients, suggesting that an intracellular bacterial population is relevant during human infection [107]. Intracellular bacteria are protected from both antibiotic treatment and clearance by extracellular immune factors, including complement, antibodies, and phagocytosis. These bacteria may therefore serve as a reservoir of antibiotic-recalcitrant bacteria, potentially leading to recurrent and chronic infections. However, it should be noted that intracellular *P. aeruginosa* manifests a somewhat patchy distribution in patient lung samples. For example, they were detected in only three out of seven lung samples from end-stage CF patients, and even then, were associated with only 0.01–0.5% of epithelial cells in the samples [107,108]. Mitigating, lung explants from end-stage CF lung disease are probably not representative of *P. aeruginosa* infections in most CF patients.

The intracellular lifestyle of *P. aeruginosa* involves two steps: internalization and intracellular survival. Internalization depends on the interaction between various bacterial adhesins and receptors on the host cell surface followed by “hijacking” of host cell internalization machinery [109]. The presence of host cell factors could influence which cell types support *P. aeruginosa* internalization. Class II (T3S) virulence factors have been shown to be cytotoxic and to block bacterial internalization; given that many *P. aeruginosa* isolates are T3S^+^, this is difficult to reconcile with an intracellular bacterial lifestyle. However, the expression of the T3S system is heterogenous and it has been suggested that T3S^−^ bacteria are more likely to be internalized [109]. Within the cell *P. aeruginosa* can reside either in membrane-bound vacuoles or in the cytoplasm. To survive in vacuoles, bacteria must avoid lysosomal acidification and killing, whereas cytoplasmic bacteria must avoid cell-autonomous immune factors, including bacterial autophagy and host cell death [110]. The ability of *P. aeruginosa* to survive these challenges suggests it has adapted to intracellular survival. T3S system virulence factors have been implicated in reducing vacuole acidification, promoting vacuolar escape, inhibiting autophagy and in preventing host cell death [111]. However, the exact mechanisms mediating these actions remain to be understood.

There is also variability in the ability of different *P. aeruginosa* strains to survive intracellularly. This may be partially explained by the balance between T3S factors that promote cell lysis and those that enable intracellular survival. The situation is further complicated by the finding that different *P. aeruginosa* clinical isolates can exhibit comparable internalization rates *in vitro* yet display significant differences in intracellular survival and host cell cytotoxicity [106]. This variability has been postulated to give rise to host-adapted intracellular *P. aeruginosa* strains that promote chronic infections.

## Biofilms: virulent or not?

Over the last few years, it has become axiomatic that planktonic cultures of *P. aeruginosa* express high levels of virulence factors and are associated with acute infection, whereas biofilms of the organism manifest much lower virulence and are associated with chronic infection. Excitingly, recent evidence suggests that under some conditions, the switch between these phenotypes is regulated by a small RNA, designated sicX [112]. However, and as noted elsewhere, distinctions are rarely as black-and-white as this. We now know that, like their planktonic counterparts, the cells in *P. aeruginosa* biofilms are secretion-competent and release copious quantities of protein into the surrounding extracellular milieu [67,113,114]. In order to escape the biofilm, these secreted proteins must pass through the polysaccharide matrix; a “bioglue” that encapsulates the cells to hold the assemblage together. Although these polysaccharides are not “traditional” virulence factors in their own right, they are often classified as virulence factors due to their ability to protect the cells from immune surveillance and clearance. The matrix also acts to protect embedded cells from antimicrobial agents, and the failure of these drugs to penetrate the biofilm, combined with the modified metabolic activity of cells within the biofilm, leads to antibiotic minimum inhibitory concentrations up to 10,000 times higher than associated with planktonic cells [115]. This makes eradication of biofilm-associated infections particularly challenging.

## The influence of other microbes on *P. aeruginosa* virulence

As with biofilms, it is becoming axiomatic (although not strictly true in all circumstances) to consider chronic infection scenarios as being polymicrobial [116]. This, in turn, raises the question of how – if at all – the presence of other microbes influences the virulence of *P. aeruginosa*. This is a difficult question to address for several reasons. First, there have been significant challenges associated with stably co-culturing *P. aeruginosa* with other microbes, although progress has been made [117–121] and it is clear that the presence of other species profoundly affects phenotypes such as antimicrobial resistance [122] and virulence [123]. Second, disentangling the effects of co-habiting microbiota on the host (e.g., host immune response) from the effects on individual species comprising the microbiota can be challenging. Finally, although several media have been developed for such studies (e.g., burn wound medium [26], chronic wound medium [124], and artificial sputum medium [125]), most of these have been optimized for *P. aeruginosa* alone, rather than for *P. aeruginosa* and potential co-habitants. Moreover, even minor alterations in the medium composition can have a large (and usually, unintended) impact on virulence [125]. Awareness of this is important, since human-derived sputum has a large impact on QS, and therefore, potentially also virulence [126].

The complications above aside, it is now clear that *P. aeruginosa* senses and responds to signals and cues released by co-habiting species. For example, *Staphylococcus aureus*-derived GlcNAc enhances the PQS-dependent production of elastase and pyocyanin by *P. aeruginosa* [123]. Similarly, it is known that *Candida albicans*, a common airway co-habitant, releases a compound (farnesol) that can “jam” PQS-dependent QS in *P. aeruginosa* [127] (although to complicate things, farnesol impinges on host metabolism too [128], and can also stimulate PQS production in *P. aeruginosa lasR* mutants [129]). Of course, there is a two-way direction of travel here, and *P. aeruginosa*-derived compounds can also impact on the virulence of nearby microbial species; the appearance of small colony variants of *S. aureus* is stimulated by the alkylquinolone HQNO [130], and alkylquinolone derivatives have recently been shown to have an impact on virulence phenotypes of several ESKAPE pathogens [131].

## Concluding comments and vistas for future study

Our goal in this review was not to simply go over material that has been recently reviewed in detail by others, e.g., individual classes of virulence factor [132–134]. Instead, our aim has been to highlight poorly-developed (largely due to experimental intractability) areas of understanding that are likely to see substantial progression in the near future. One such area is the increasing realization that the global *P. aeruginosa* “pan-virulome” is likely to be huge [135], and around 12% of the identified mobilome is thought to be associated with virulence. This figure is likely an underestimate, since many more virulence factors remain to be discovered; a substantial portion of the secretome is still comprised of “hypothetical” (uncharacterized) proteins. That so many virulence factors are housed on horizontally-acquired islands begs the question of how, if at all, their expression is coordinated with that of virulence factors in the core genome. There is evidence to suggest that such “imported” virulence factors can come under the control of endogenous regulatory networks, such as QS in some other organisms [136], but to our knowledge, no similar experiments have been done for *P. aeruginosa*. Nonetheless, prediction of *P. aeruginosa* virulence profiles based on machine learning and genomic data is becoming a reality (e.g., [137]). Although this will be of great use at a clinical level, as always, the problem with ML- and AI-based approaches is that they provide an answer without explaining how the answer was derived: they are less informative in terms of basic biological insight.

Another area likely to see development is our understanding of “unconventional” molecules as mediators of virulence. For example, little is known about how, if at all, transmissible RNA (delivered directly into host cells or adjacent bacterial cells, for example) plays a role in *P. aeruginosa* virulence. Transmissible RNA has been recognized as a potential virulence determinant in other biological systems [138]. The biofilm-associated “matrixome” also looks set to yield many new insights, especially if the matrix does indeed prove (as seems likely) to be a distinct extracellular compartment. Perhaps most exciting though are the insights to be gained in looking at how other species influence *P. aeruginosa* virulence (and *vice versa*), since this is an experimental door that has only recently opened, allowing access to a good deal of novel biology. A similar knowledge gap relates to the extent to which the host plays any role in infection outcomes. For example, it is possible that some strain-dependent differences in “virulence” may actually reflect the host response rather than any intrinsic trait(s) of the infecting organism. In this scenario, “highly virulent” isolates may trigger the host immune system in a way that is different from less-virulent strains. Finally, and with a knowledge gap in our evolutionary understanding of infection, we note that although T3S is a cheatable trait, less is known about cheats affecting T6S – we would predict that these cheats do arise, although little is currently known about this.

In a related vein, we also note that the population of any given species, even in a multi-species chronic infection, is itself usually heterogenous. Thus, in a given CF sputum sample, we may find [clonally derived] auxotrophs, QS mutants, strains displaying AMR, small colony variants, altered protease and/or siderophore production, hypermutability, and so on [139,140]. Assuming a similar intra-species heterogeneity for each of the other co-habiting species in the niche (and this supposition has yet to be verified), the number of contributing factors that might influence *P. aeruginosa* trajectories in an infection scenario increases exponentially.

## Data Availability

There are no data associated with this review article.
